# Methicillin-Resistant Staphylococcus aureus in Urinary Tract Infections: A Comprehensive Review With Insights From a North Indian Cohort

**DOI:** 10.7759/cureus.99015

**Published:** 2025-12-11

**Authors:** Gaurav Kumar, Snehanshu Shukla, Sheetal Agarwal, Deepika Shukla, Swetha R Soni, Rajesh K Verma, Vidushi Singh, Awadhesh Kumar, Deepak Kumar

**Affiliations:** 1 Department of Microbiology, Rajarshi Dashrath Autonomous State Medical College, Ayodhya, Ayodhya, IND; 2 Department of Microbiology, King George's Medical University, Lucknow, IND; 3 Department of Microbiology, Maharana Pratap Dental College and Hospital, Kanpur, IND; 4 Department of Pediatric Surgery, All India Institute of Medical Sciences, Nagpur, Nagpur, IND; 5 Department of Microbiology, Uttar Pradesh University of Medical Sciences, Etawah, IND; 6 Department of Microbiology, Sanjay Gandhi Postgraduate Institute of Medical Sciences, Lucknow, IND; 7 Department of Microbiology, Mahamaya Rajkiya Allopathic Medical College, Akbarpur, IND

**Keywords:** antimicrobial resistance, india, meca, mrsa, urinary tract infection, vancomycin

## Abstract

Methicillin-resistant *Staphylococcus aureus* (MRSA) remains a major global public health concern, with urinary tract infections (UTIs) representing a less common but clinically important manifestation. Although Gram-negative bacteria remain the predominant uropathogens, increasing MRSA detection in urinary isolates presents diagnostic and therapeutic challenges, particularly in high-risk groups such as catheterised or hospitalised patients. This review consolidates global evidence published between 2000 and 2025, focusing on the epidemiology, molecular characteristics, antimicrobial resistance, diagnostics, and management of MRSA UTIs. Reported prevalence ranges from 2% to over 10%, varying with geography and patient factors. Key virulence mechanisms include urine-induced gene expression, copper resistance, metabolic adaptability, and biofilm formation, which enhance persistence and treatment failure risk. Resistance to β-lactams, fluoroquinolones, and aminoglycosides is widespread, whereas vancomycin and linezolid remain reliably effective. Advances in molecular diagnostics, such as PCR and sequencing, have improved detection accuracy and supported antimicrobial stewardship. MRSA, though relatively uncommon, poses significant risks to vulnerable populations and exemplifies broader global antimicrobial resistance trends. Strengthened surveillance, rapid diagnostics, judicious empiric therapy, and stewardship programs, integrated within a One Health framework, are essential to reduce the growing burden of MRSA urinary infections worldwide.

## Introduction and background

Urinary tract infections (UTIs) are among the most prevalent bacterial infections globally, affecting both community and hospital populations and contributing significantly to morbidity, healthcare costs, and antibiotic consumption [1]. While *Escherichia coli* and other Enterobacterales remain the predominant uropathogens, recent evidence highlights a rising role of Gram-positive organisms, notably *Staphylococcus aureus *[2]. This trend has gained clinical importance with the emergence of methicillin-resistant *S. aureus *(MRSA) as a uropathogen, which, although historically rare, is now increasingly encountered in both community and healthcare settings [3].

The epidemiology of MRSA urinary infections is influenced by several risk factors. Indwelling catheterisation, urological abnormalities such as renal calculi or hydronephrosis, and comorbid conditions like diabetes mellitus predispose to *S. aureus* colonisation and infection [4]. Furthermore, the rise in healthcare-associated infections and widespread use of invasive devices has expanded MRSA’s ecological niche [5]. Catheter-associated UTIs (CAUTIs) not only prolong hospital stays and increase costs but can also lead to bacteremia and higher mortality when left untreated [6]. The detection of *S. aureus* in urine may additionally indicate systemic infection, underscoring the need for early recognition and targeted therapy [3].

Clinically, MRSA urinary infections can present with both typical and atypical features. While symptoms such as dysuria, frequency, and suprapubic pain are common, MRSA infections may manifest with systemic involvement or abscess formation [2,7]. Severe complications, including renal or prostatic abscesses, have been reported even in immunocompetent individuals, illustrating diagnostic challenges and significant morbidity [5,8]. The increasing detection of MRSA in urinary isolates reflects the broader global challenge of antimicrobial resistance (AMR) [9]. Resistance determinants such as mecA compromise β-lactam efficacy and limit therapeutic options in resource-constrained settings [3,10]. Clinicians must therefore balance adequate empiric coverage with stewardship principles, as both undertreatment and overuse of broad-spectrum agents can lead to poor outcomes and further resistance [9,11].

Published evidence from South Asia demonstrates variable MRSA prevalence among urinary isolates, reflecting geographical and methodological diversity [6,12]. However, comprehensive surveillance and synthesis of regional data remain limited. Therefore, this review aims to integrate global and regional literature to provide a consolidated understanding of the epidemiology, pathogenesis, diagnostics, and clinical management of MRSA UTIs, situating them within the broader context of AMR.

## Review

Methodology

This narrative review synthesised published evidence on MRSA UTIs without the intention of performing a formal systematic review between 2000 and 2025. A structured but non-systematic search of PubMed, Scopus, and Google Scholar was conducted using combinations of the terms ‘MRSA’, ‘urinary tract infection’, ‘bacteriuria’, ‘epidemiology’, and ‘antimicrobial resistance’. Studies were included if they reported clinical, epidemiological, diagnostic, or therapeutic aspects of MRSA UTIs in humans. No restrictions were placed on study design or geographical region due to the limited availability of data. Relevant reference lists were also manually screened to identify additional studies. This review did not perform statistical pooling, meta-analysis, or quantitative synthesis. Any meta-analyses cited were reported descriptively to contextualise existing evidence, and no independent statistical methods were applied in the preparation of this narrative review.

Epidemiology and clinical spectrum of MRSA UTIs

The burden of MRSA in UTIs has been reported to increase across regions, although prevalence rates vary significantly according to geography, patient population, and study methodology. An early report showed that MRSA represents a measurable proportion of UTI cases in sub-Saharan Africa and the Horn of Africa [6]. In Ethiopia, a hospital-based cross-sectional study demonstrated that MRSA constituted a substantial percentage of *S. aureus* urinary isolates, with prior antibiotic exposure and hospitalisation identified as major risk factors [10]. These findings highlight the occurrence of MRSA in UTIs even in resource-limited settings, where diagnostic capabilities are scarce and empiric treatment is often based on syndromic rather than microbiological confirmation [11].

Building on these regional observations, meta-analyses provide a valuable broader perspective on the epidemiology of MRSA in urinary infections. A systematic review and meta-analysis examining *S. aureus* UTIs confirmed their occurrence across diverse regions and patient groups [12]. The pooled prevalence varied but consistently demonstrated that MRSA constituted a small yet meaningful subset of urinary infections. Importantly, these infections were associated with a higher risk of treatment failure when empiric regimens lacked MRSA coverage [4], underscoring the epidemiological and clinical relevance of accurate risk stratification and diagnostic precision.

**Figure 1:**
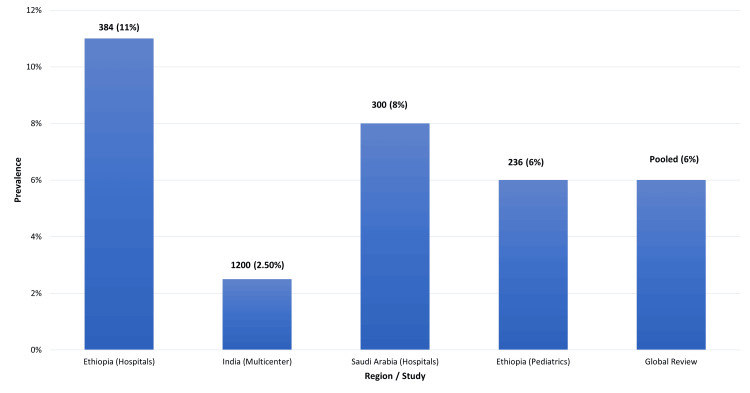
Global Epidemiology of MRSA in UTIs MRSA: methicillin-resistant *Staphylococcus aureus*; UTIs: urinary tract infections Image credit: Deepak Kumar; adapted from references [6,10-13].

In South Asia, the epidemiology of MRSA UTIs reflects both hospital and community trends [13]. Data from India have underscored the importance of resistance surveillance across diverse biological niches. A recent investigation focusing on donor human milk found the presence of *Staphylococcal* isolates harbouring mecA, the gene conferring methicillin resistance [11]. Although this study did not focus specifically on urinary samples, it illustrates the widespread circulation of resistant *Staphylococci* in environments relevant to neonates and maternal health, reinforcing concerns about possible transmission pathways. Such findings underscore that, although this example does not represent a classical One Health interaction linking human, animal, and environmental health, it nevertheless illustrates how resistant staphylococcal populations present in non-urinary reservoirs may contribute to the risk of subsequent colonisation and infection [13]. Global prevalence patterns of MRSA in urinary isolates are summarised in Figure 1.

Mechanistic insights into resistance also contribute to understanding the epidemiology of MRSA in urinary infections [14]. Structural modelling studies on cefoxitin resistance in MRSA provide clarity on the molecular basis for diagnostic screening and therapeutic failure [12]. These mechanistic data align with surveillance reports that consistently detect cefoxitin resistance as a marker for methicillin resistance, further cementing the role of this antibiotic in routine phenotypic testing for MRSA in both urinary and other clinical isolates [15].

Globally, AMR trends frame the context in which MRSA UTIs are evaluated [16]. A review of conventional and novel antibiotics has highlighted the limitations of existing therapeutic options against multidrug-resistant pathogens, including MRSA [9]. The restricted efficacy of older agents and the uneven availability of novel antimicrobials mean that local epidemiology has direct therapeutic implications [17]. This is particularly relevant in urinary infections, where narrow empiric regimens may fail if MRSA prevalence is underappreciated.

Specific reports of resistant MRSA in non-urinary infections also help illuminate the epidemiological landscape [18]. For example, clinical isolates of β-lactam-resistant *S. aureus* recovered from cases of spinal osteomyelitis and spondylodiscitis show the breadth of resistant strains across different infection sites [10]. Although not urinary in origin, these isolates represent the same clonal resistance mechanisms found in MRSA UTIs and demonstrate the systemic potential of such organisms to cause disease in multiple organ systems [19].

The zoonotic and foodborne dimension of MRSA epidemiology also merits attention. A molecular surveillance study of MRSA isolates from retail raw milk in China demonstrated genetic relatedness to strains from human patients, suggesting cross-species evolution [11]. Such findings indicate that reservoirs of MRSA outside hospitals and communities may serve as reservoirs for urinary infections, particularly in agricultural societies with close animal contact [20]. They further highlight that urinary isolates should not be considered in isolation but rather as part of a broader transmission web.

Mortality outcomes provide another critical dimension to the epidemiological picture. A systematic review and meta-analysis evaluating empirical MRSA coverage demonstrated increased mortality in patients when early empirical coverage was absent [12]. Although this study synthesised data from a wide range of MRSA infections, the findings apply to urinary infections as well, given the potential for bacteremic progression from urinary foci [21]. These results reinforce the importance of situating MRSA UTIs not only as localised infections but also as potential precursors to systemic, life-threatening disease [22].

The Middle East provides additional epidemiological data that informs the understanding of MRSA UTIs. A study conducted in western Saudi Arabia identified the prevalence, genotypes, and molecular relatedness of MRSA isolates from hospitalised patients [14]. This study revealed significant clonal diversity among MRSA isolates, reflecting both local transmission dynamics and possible importation of strains through global travel and labour migration [23]. For urinary infections, this genetic heterogeneity means that clinicians may face varied resistance patterns even within a single health system. The reported prevalence of MRSA in urinary isolates varies significantly across regions and populations. In Ethiopian hospitals, MRSA accounted for approximately 10-12% of UTI cases [6], while Indian multicenter studies reported lower rates, around 2-3% [24]. In Saudi Arabia, MRSA prevalence among urinary isolates was 7-9% [14]. Additional data suggest zoonotic reservoirs, such as dairy cow strains in Africa [13], and the emergence of *Staphylococcus argenteus* in pediatric infections [15]. These findings are summarised in Table 1.

**Table 1:** Global Prevalence of MRSA in Urinary Isolates MRSA: methicillin-resistant *Staphylococcus aureus*; UTIs: urinary tract infections

Region/Country	Study Population	MRSA Prevalence in UTIs	References
Ethiopia	Hospital patients	10-12%	[6]
India (multicenter)	General UTI patients	2-3%	[24]
Saudi Arabia	Hospital isolates	7-9%	[14]
Dairy cow strains (zoonotic risk, context), Africa	Veterinary isolates	MRSA found in milk, with potential spread to humans	[13]
Pediatric infections (*Staphylococcus argenteus*), China	Children with UTIs/infections	Rare but emerging	[15]

Similarly, agricultural settings in Ethiopia have provided insight into MRSA virulence and resistance profiles. A study of *Staphylococcus *isolates from mastitic dairy cows identified multiple virulence genes and resistance traits [13]. While not urinary in origin, these findings demonstrate how animal reservoirs can harbour strains with potential for human infection [25]. Such zoonotic reservoirs may play an underappreciated role in the epidemiology of MRSA urinary infections, particularly in rural communities where human-animal interactions are frequent [26].

Emerging pathogens within the *Staphylococcal* genus also deserve consideration.* S. argenteus*, a close relative of *S. aureus*, has been identified in pediatric infections in East China [15]. Although the infections caused by it are mostly researched in bloodstream and skin infections, its clinical resemblance to MRSA makes it possible that it is underdiagnosed in urinary infections. The development of *S. argenteus* demonstrates the fluidity of *Staphylococcus* epidemiology, in which new species can confound both diagnosis and treatment patterns [27].

Collectively, epidemiological studies show that MRSA UTIs represent a small but increasing proportion of all urinary infections. The prevalence rates are variable and depend on patient comorbidities, regional antibiotic consumption, and the existence of local reservoirs in animals and the environment [28]. In addition, the clinical range of MRSA UTIs extends from simple cystitis to invasive pathologies, such as prostatic abscesses and bacteremia [20]. The adverse mortality consequences of ineffective empiric coverage highlight the clinical significance of even low-prevalence MRSA UTIs [12]. It is in this background that regional surveillance across South Asia is critical in putting global epidemiological trends into perspective to inform evidence-based clinical practice [24].

Pathogenesis, virulence, and molecular characteristics of MRSA in UTIs

The capability of MRSA to cause infection in the urinary tract is a complex interrelationship between host factors, bacterial virulence determinants, and adaptive metabolic pathways [2]. Contrary to Gram-negative bacilli, which employ uropathogenic adhesins and toxins, *S. aureus* utilises distinct mechanisms that enable its persistence and growth in the urinary environment [3,16]. Recent investigations have emphasised that urine alone can modify MRSA phenotype and virulence, establishing a dynamic interaction between pathogen and host [16,29].

A landmark experimental study demonstrated that exposure to human urine significantly alters the MRSA transcriptome by upregulating virulence-related genes [16]. This finding indicates that the urinary milieu actively modulates bacterial gene expression, enhancing MRSA’s potential to cause symptomatic infection. Clinically, this adaptability may explain why MRSA urinary colonisation occasionally progresses to invasive disease, especially among patients with indwelling catheters or comorbidities such as diabetes or renal dysfunction [20].

Metal ion regulation further contributes to MRSA urinary pathogenesis. Copper, an essential yet potentially toxic ion, was shown to enhance MRSA fitness in a murine model of UTI [17]. The genetic determinants of copper resistance provide MRSA with an adaptive advantage in the urinary tract environment, where metal ion concentrations fluctuate due to diet or disease states [29]. These insights also open avenues for exploring metal modulation as a non-antibiotic therapeutic target [17].

Metabolic adaptability plays an equally vital role in MRSA persistence. Experimental investigations into central carbon metabolism and mannitol utilisation revealed that these pathways are indispensable for MRSA colonisation of urinary catheters and infection establishment [18]. Disruption of mannitol metabolism significantly reduced bacterial adherence and biofilm formation, supporting the hypothesis that metabolic flexibility underpins chronic infection potential [30]. Integrative genomic analyses of biofilm-forming MRSA strains have confirmed a strong overlap between metabolic and virulence gene networks, reinforcing their synergistic role in infection persistence [31].

**Figure 2:**
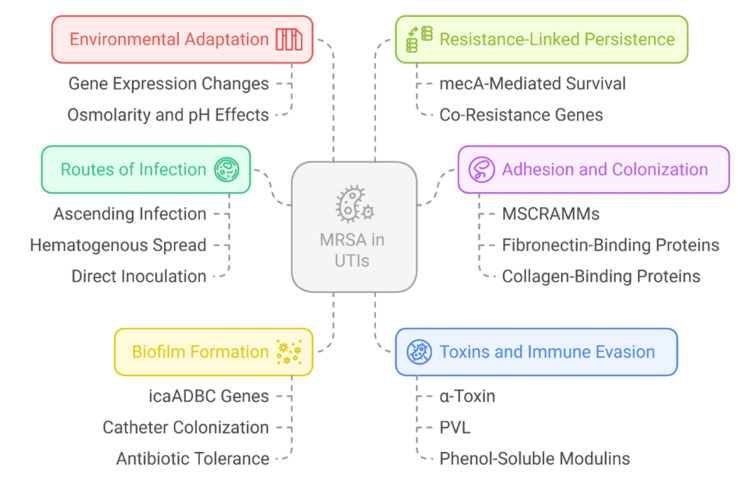
Pathogenesis of MRSA in Urinary Tract Infections (UTIs) icaADBC: intercellular adhesion gene cluster; MSCRAMMs: microbial surface components recognising adhesive matrix molecules; PVL: Panton-Valentine leukocidin; MRSA: methicillin-resistant *Staphylococcus aureus* Image credits: Deepak Kumar

Collectively, these findings illustrate that MRSA’s success in the urinary tract depends on a combination of transcriptional adaptation, metal ion regulation, and metabolic reprogramming [32]. Understanding these multifactorial mechanisms not only clarifies MRSA pathogenesis but also highlights novel molecular targets for prevention and treatment strategies. The most important aspects of MRSA pathogenesis in UTIs, such as routes of infection, virulence determinants, and host-pathogen interactions, are outlined in Figure 2.

The host immune response also shapes the pathogenesis of MRSA in UTIs. Investigations into inflammasome pathways revealed that the NLRP3 inflammasome, a critical mediator of innate immunity, is dispensable in MRSA UTIs [19]. This finding indicates that MRSA may bypass or subvert specific host immune defences, contributing to persistence in the urinary tract. The absence of a protective role for NLRP3 suggests that other immune mechanisms, perhaps neutrophil recruitment or adaptive responses, may be more central in controlling MRSA urinary infections [33].

Clinically, MRSA urinary infections can present with severe complications when host-pathogen dynamics favour uncontrolled growth. For example, MRSA renal abscesses have been reported in adolescents, sometimes unmasking underlying conditions such as HIV infection [33]. Such cases highlight the intersection between bacterial virulence, host immune status, and clinical outcome. These cases illustrate how MRSA UTIs may serve as sentinel infections, signalling underlying immunosuppression or systemic vulnerability. Building on these clinical insights, molecular epidemiology defines the resistance profile and transmissibility of MRSA strains.

Beyond pathogenesis, the molecular characteristics of MRSA define its resistance profile and transmissibility. Molecular epidemiology has documented the dissemination of β-lactam-resistant *S. aureus* between humans and animals [21]. Genotypic analyses confirm that resistance determinants such as blaZ and mecA circulate across hosts, raising concerns about zoonotic transmission. For UTIs, this means that exposure to colonised animals or contaminated food products could seed resistant strains in human populations [34].

Community and environmental reservoirs further contribute to the molecular diversity of MRSA [35]. A study examining *Staphylococcal *isolates from daycare centres identified multiple antibiotic resistance and virulence genes, demonstrating how community settings serve as hubs for dissemination [22]. These findings reinforce the importance of surveillance beyond hospital settings. Children in daycare centres can serve as asymptomatic carriers, with potential downstream consequences for urinary infections when colonisation seeds invasive disease [36].

At the molecular level, the mecA gene remains the defining feature of methicillin resistance [3,7]. Its protein product, penicillin-binding protein 2a (PBP2a), confers reduced affinity for β-lactam antibiotics, rendering standard therapies ineffective [3,7]. Variants of mecA, as well as alternative resistance genes such as mecC, expand the repertoire of resistance mechanisms. Additionally, the staphylococcal cassette chromosome mec (SCCmec) element provides a mobile genetic platform facilitating horizontal transfer across strains and species [3,7]. This genomic plasticity explains the widespread and persistent nature of methicillin resistance across diverse clinical niches, including the urinary tract [24]. Urinary MRSA exhibits a variety of virulence traits, including copper resistance, metabolic flexibility, and NLRP3 inflammasome modulation (Table 2).

**Table 2:** Virulence and Molecular Features of MRSA UTIs MRSA: methicillin-resistant *Staphylococcus aureus*; UTIs: urinary tract infections

Virulence Factor/Mechanism	Description	References
Urine-induced transcriptomic shifts	Alters gene expression to adapt to the urinary environment	[16]
Copper resistance mechanisms	Enhances fitness and persistence in UTIs	[17]
Mannitol utilization	Supports metabolic flexibility and survival	[18]
NLRP3 inflammasome modulation	Influences the host's immune response	[19]
Abscess formation (renal/prostatic)	Associated with invasive disease	[33]
Zoonotic gene transfer	Transmission from livestock or the environment	[21]
Resistance genes in childcare/daycare settings	MRSA circulation beyond hospitals	[22]

Collectively, the pathogenesis and molecular characteristics of MRSA in UTIs highlight a multifaceted challenge. Urine alters bacterial gene expression, copper and metabolic adaptations promote survival, and the organism exploits host immune gaps to persist [16-19]. Clinically, these features manifest as infections that may transition from colonisation to invasive disease, with severe outcomes in immunocompromised hosts [20,33]. Molecular evidence further demonstrates that resistance determinants are not confined to hospitals but circulate widely in communities, animals, and the environment [21,37]. Understanding these complex dynamics is essential for developing targeted therapies and infection control strategies, especially as the urinary tract emerges as an important, though less common, site of MRSA disease [3].

AMR trends in MRSA UTIs

Novel and Emerging Agents Against MRSA

The search for new therapeutic agents has produced promising results, though challenges remain in their clinical application. Biapenem, a novel carbapenem, has shown in vitro activity against resistant Gram-positive organisms, including MRSA [23]. Although carbapenems have traditionally been ineffective against methicillin-resistant strains due to mecA-encoded PBP2a, structural modifications in newer molecules suggest potential avenues for future therapy [3]. However, clinical translation of these findings remains limited, and biapenem is not currently a standard therapy for MRSA urinary infections. In addition, other established and emerging agents, such as daptomycin and ceftaroline, as well as newer lipoglycopeptides including dalbavancin and oritavancin, have demonstrated activity against MRSA but present important limitations in the context of urinary infections, including restricted clinical data, variable pharmacokinetic profiles, and, in some cases, inadequate urinary tract penetration similar to agents such as tigecycline.

South Asia, in particular, exemplifies the need for new agents. A multi-institutional antibiogram study revealed high rates of multidrug resistance in *S. aureus* isolates, including urinary pathogens [24]. Resistance was not confined to β-lactams but extended to commonly used oral and intravenous agents, complicating empiric therapy; this is particularly important because several oral agents with activity against MRSA, such as doxycycline and cotrimoxazole, remain valuable options for treating MRSA UTIs when susceptibility is preserved [35]. Such findings are especially concerning in regions where novel antimicrobials like biapenem or newer oxazolidinones may be unavailable due to cost or regulatory barriers [24].

Resistance in Pediatric and Community Settings

MRSA resistance epidemiology is not restricted to adults. Children, including neonates and school-age groups, are also at high risk. A multicentric study in Ethiopia evaluating bacterial uropathogens in children revealed *S. aureus* as a contributor to urinary isolates that were mostly methicillin-resistant [25]. The existence of resistant strains in children is of particular concern because of the treatment limitations in this vulnerable population, where there are narrow therapeutic margins and few pediatric-approved antibiotics that restrict clinical options [38].

Community-acquired MRSA (CA-MRSA) adds another layer of complexity. In contrast to hospital-acquired strains, CA-MRSA is usually susceptible to some non-β-lactam antibiotics but can develop resistance over time. The growing rate of CA-MRSA strains among urinary isolates implies a loss of epidemiologic distinctions, as strains previously confined to hospitals now circulate in the community and vice versa [35]. This evolving resistance pattern presents a growing challenge for clinicians, particularly in outpatient settings where UTI management relies on empiric antibiotic selection [39].

Stewardship and Infection Prevention Guidelines

Robust stewardship measures are essential to curb the rise of resistance. The 2022 Society for Healthcare Epidemiology of America (SHEA) compendium update highlighted infection prevention as part and parcel of antimicrobial stewardship [26]. In the case of MRSA UTIs, not only should prudent antibiotic use be observed, but also preventive measures, including the minimisation of catheter use, diagnostic accuracy, and the enhancement of hand hygiene. Colonisation and infection prevention decrease exposure to antimicrobials, which indirectly reduces resistance, and also lowers the likelihood of subsequent colonisation by resistant organisms.

The clinical outcome of antimicrobial therapy may also influence resistance patterns. A case report demonstrated serious toxicity when using sulfamethoxazole-trimethoprim (Bactrim) to treat a patient with *Staphylococcus *infection [27]. Although not specific to urinary isolates, it highlights the clinical challenge when therapeutic options are limited. Adverse drug events can also discourage the use of otherwise effective agents, leaving fewer alternatives and promoting the reliance on broader-spectrum antibiotics that accelerate resistance.

Infection Control Policies and Resistance Outcomes

MRSA prevalence and resistance patterns are greatly affected by institutional infection control policies. A six-year time-series study has shown no increase in infection rates in the event of discontinuing contact precautions against MRSA when combined with robust horizontal measures (universal decolonisation and hand hygiene) [28]. These findings imply that blanket isolation policies might not be essential in case there is a strong infection prevention strategy in place. In MRSA UTIs, especially in catheterised patients, focused infection control strategies may be more effective at reducing risk compared to blanket isolation practices, as well as lowering patient stigma and the burden of resources [28].

Virulence and Resistance Intersections

Biofilm formation is one of the key interactions between virulence and AMR in MRSA. A genomic study of biofilm-forming MRSA isolates identified a complex interrelationship between resistance and virulence genes [29]. Biofilms in the urinary tract are of particular concern because they underlie catheter-associated infections and often drive persistence despite appropriate antibiotic therapy [35]. Once established, biofilms protect the bacterial community from host immune defences and create microenvironments conducive to horizontal gene transfer [35]. This enhances the dissemination of resistance genes within the urinary tract, potentially leading to recurrent or relapsing infections.

Synthesis of trends

The resistance patterns in MRSA urinary infections have several defining themes. First, the urinary tract mirrors broader global trends in AMR, as multidrug resistance rates are high in urinary, bloodstream, and respiratory infections [34]. Second, pediatric and community environments are increasingly involved, making it untenable to assume that MRSA UTIs are confined to hospitalised adults [35]. Third, infection control and stewardship policies remain central to limiting resistance, and targeted strategies have demonstrated greater effectiveness than universal isolation measures [28]. Lastly, virulence mechanisms such as biofilm formation overlap with resistance, compounding the clinical burden of MRSA UTIs [29].

Diagnostics of MRSA in UTIs

Conventional Microbiological Approaches

Standard culture techniques remain the first-line diagnostic method for UTIs, including those caused by MRSA. Midstream urine samples, catheterised specimens, or suprapubic aspirates are cultured on selective media, followed by identification of *S. aureus* using biochemical assays [40]. Methicillin resistance is typically inferred through cefoxitin disk diffusion testing, which serves as a reliable surrogate for mecA-mediated resistance and offers superior performance compared with older oxacillin assays [8]. However, phenotypic tests may yield ambiguous results in the presence of heteroresistance or borderline susceptibility, necessitating confirmatory methods [7].

While culture-based diagnostics are inexpensive and widely available, their turnaround time of 24-48 hours may delay initiation of targeted therapy. This diagnostic lag often forces clinicians to rely on empiric regimens that may not provide adequate MRSA coverage, increasing the risk of disease progression or the unnecessary use of broad-spectrum antibiotics that drive resistance [6,41].

Molecular Diagnostic Advances

Advances in molecular diagnostics, including polymerase chain reaction (PCR) and gene-based assays, enable rapid and precise detection of mecA and other resistance determinants directly from urine or cultured isolates. These technologies markedly shorten the diagnostic window, facilitate early initiation of targeted therapy, and support antimicrobial stewardship. Molecular testing also differentiates MRSA from methicillin-susceptible *S. aureus* (MSSA), guiding clinicians toward appropriate antimicrobial selection [31].

Stewardship and Diagnostic Integration

The diagnostic landscape is closely linked to antimicrobial stewardship. A study evaluating MRSA PCR-guided de-escalation in pneumonia demonstrated that rapid exclusion of MRSA reduced unnecessary exposure to broad-spectrum agents without compromising clinical outcomes [31]. Although these findings arise from respiratory infections, which differ substantially from lower or CAUTIs in pathophysiology and urgency, they nevertheless illustrate how rapid MRSA rule-out testing can support judicious antibiotic use when appropriately interpreted. Importantly, interpretation remains challenging in the absence of culture confirmation, particularly in patients with indwelling catheters, where distinguishing colonisation from true infection is difficult.

For urinary infections, stewardship strategies may include reflex PCR testing for suspected MRSA cases or molecular screening in patients with prior MRSA colonisation. Integrating rapid diagnostics into clinical workflows reduces reliance on empiric vancomycin or linezolid, preserving these agents for patients who genuinely require them [31].

Environmental and Reservoir Surveillance

Environmental diagnostics provide insight into reservoirs of AMR. Genetic characterisation of staphylococci from water sources has revealed methicillin resistance and virulence determinants [32]. These findings highlight the circulation of MRSA beyond hospitals and human carriers. For urinary infections, such reservoirs underscore the potential for community acquisition from nontraditional sources.

Environmental surveillance aligns with the One Health perspective, emphasising interconnected human, animal, and environmental health. Detection of multidrug-resistant staphylococci in water, livestock, and food products contributes to a broader understanding of MRSA transmission pathways, informing both public health and clinical strategies [42,43].

Emerging Diagnostic Technologies

Although PCR dominates current molecular testing, newer technologies, including loop-mediated isothermal amplification (LAMP), CRISPR-based diagnostics, and next-generation sequencing (NGS), offer promise for rapid, point-of-care detection of MRSA in urine. These approaches aim to shorten turnaround time, increase sensitivity, and enable multiplex detection of pathogens and resistance markers. However, widespread adoption remains limited by cost, validation requirements, and laboratory infrastructure.

Synthesis

Diagnostics for MRSA UTIs illustrate a continuum from traditional culture to advanced molecular techniques. While culture remains essential for definitive diagnosis and susceptibility testing, molecular assays offer superior speed and specificity that can transform stewardship practices. Broader adoption of PCR-based tools in urinary diagnostics promises to optimise therapy, minimise unnecessary antimicrobial exposure, and prevent progression to invasive disease. Moreover, environmental surveillance extends diagnostic insight, positioning urinary infections within a wider network of reservoirs and transmission pathways.

Treatment and clinical management of MRSA UTIs

**Figure 3:**
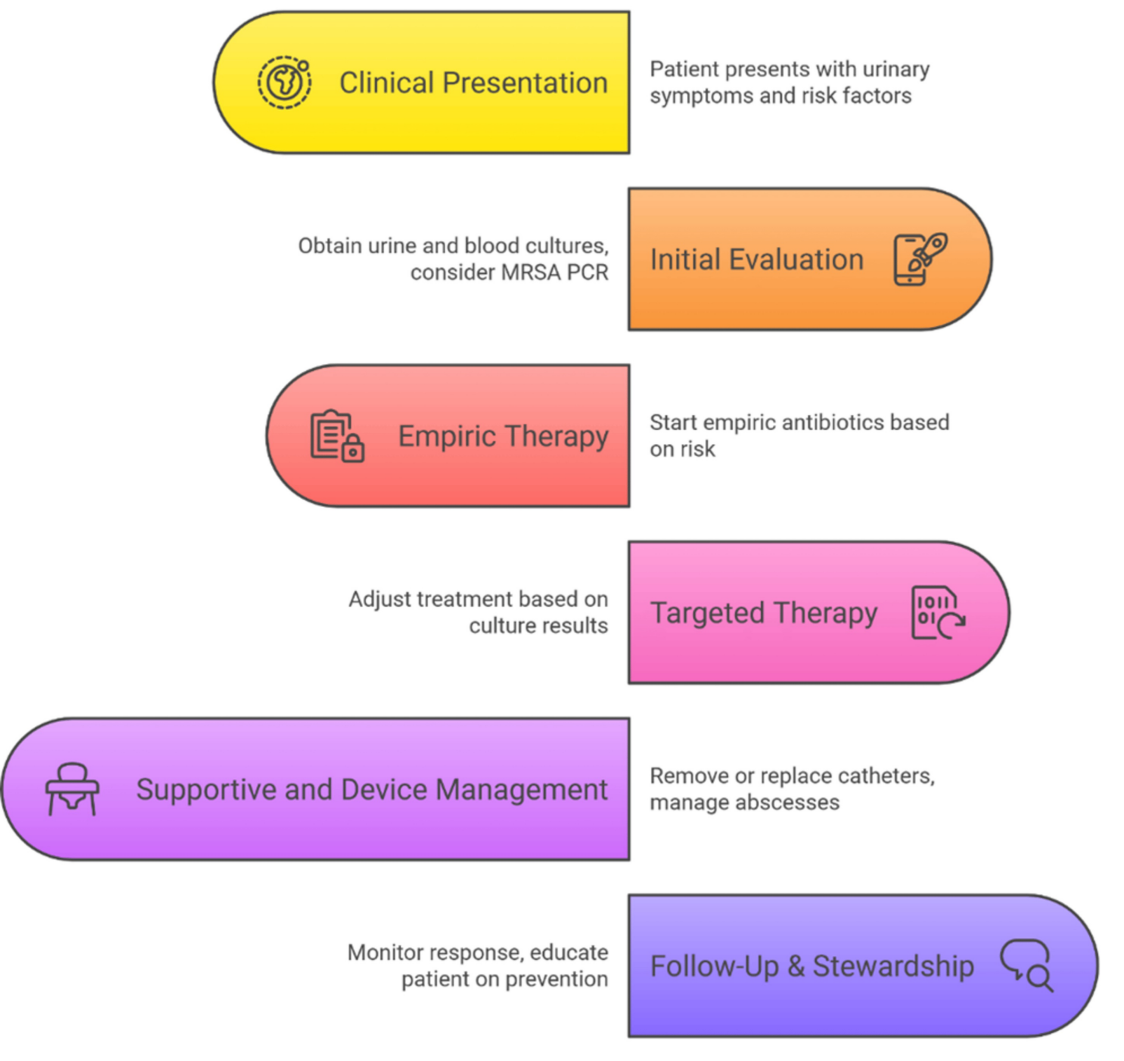
Management Framework for MRSA Urinary Infections MRSA: methicillin-resistant* Staphylococcus aureus*; PCR: polymerase chain reaction Image credits: Deepak Kumar

A practical management framework is outlined in Figure 3, emphasising the integration of rapid diagnostics with antimicrobial stewardship principles.

Clinical presentations and therapeutic implications

The spectrum of MRSA urinary infections ranges from asymptomatic bacteriuria to severe abscesses requiring surgical intervention. Case reports underscore the diversity of presentations and therapeutic needs. For example, a pediatric patient developed a prostatic abscess secondary to MRSA UTI, requiring both surgical drainage and prolonged antibiotic therapy [33]. This case highlights the importance of considering MRSA as a potential pathogen in atypical urinary presentations, particularly when conventional empiric regimens fail.

The clinical burden of MRSA infections extends beyond the urinary tract. The global burden of AMR, quantified in a large-scale analysis, identified MRSA among the leading bacterial causes of mortality worldwide [34]. Although urinary isolates constitute a minority of these cases, their contribution to systemic disease, including bacteremia originating from the urinary tract, makes MRSA UTIs clinically significant in the broader AMR landscape.

MRSA in non-urinary contexts and clinical lessons

Lessons from other infection sites inform MRSA UTI management. A meta-analysis on MRSA pneumonia emphasised the clinical consequences of delayed or absent MRSA coverage [36]. Although focused on respiratory infections, the findings offer only partial relevance to urinary infections, as pneumonia is inherently a bacteremic and time-sensitive condition, whereas many UTIs, particularly lower UTIs and CAUTIs without systemic features, do not demonstrate the same relationship between rapid antimicrobial initiation and clinical outcomes. Nevertheless, in those select UTI cases where MRSA leads to complications such as bacteremia or abscess formation, timely targeted therapy remains clinically important. These parallels support an integrated view of MRSA management across clinical syndromes.

Long-term care facilities (LTCFs) and vulnerable populations

MRSA urinary infections are particularly relevant in LTCFs and among older adults with indwelling catheters. A surveillance study demonstrated high rates of multidrug resistance among urinary pathogens in nursing home populations, including *S. aureus* [44]. These patients often present with atypical symptoms, delayed recognition, and complex comorbidities, which complicate both diagnosis and treatment. Furthermore, LTCF residents frequently receive repeated antibiotic courses, fostering resistance and narrowing therapeutic options.

Comparisons between LTCF and acute care outcomes highlight the importance of tailored management strategies. While acute care settings may enable aggressive diagnostics and intravenous therapy, LTCFs often rely on oral therapy and supportive care. This divergence underscores the necessity of novel oral agents with activity against MRSA, particularly for vulnerable populations where hospitalisation may be undesirable or impractical [45].

Therapeutic agents for MRSA UTIs

Vancomycin remains the standard intravenous therapy for severe MRSA infections, including bacteremic urinary infections. It achieves reliable activity against most MRSA isolates, though nephrotoxicity and the need for therapeutic drug monitoring limit its use. Linezolid, an oxazolidinone, offers both intravenous and oral options and is particularly useful in step-down therapy, though its hematologic toxicities preclude prolonged use, and long-term administration is further limited by neurologic adverse effects, including peripheral neuropathy and optic neuropathy. Daptomycin is another effective parenteral agent, but it requires monitoring for myopathy.

Novel and repurposed therapies are under investigation for MRSA UTIs, and several established and emerging agents, including ceftaroline and the newer lipoglycopeptides dalbavancin and oritavancin, have demonstrated activity against MRSA. However, their use in urinary infections is limited by important shortcomings, including scarce clinical data and, for some agents, suboptimal urinary tract penetration similar to that observed with tigecycline, thereby restricting their applicability in MRSA UTI management. Faropenem, a penem antibiotic, has shown promise in pediatric urinary infections, with potential application to resistant Gram-positive organisms [38]. Amniotic membrane extracts demonstrated antibacterial properties against multidrug-resistant uropathogens, including MRSA, in experimental studies [39]. Though not yet clinically applied, such alternative strategies reflect growing interest in nontraditional antimicrobials. Comparisons of acute versus LTCF settings emphasise the need for agents that balance efficacy, tolerability, and oral bioavailability [40]. These therapeutic options, though diverse, remain constrained by access, cost, and safety considerations.

Stewardship and treatment optimisation

Stewardship remains integral to MRSA UTI management. Empiric therapy should be guided by local antibiograms, with MRSA coverage reserved for patients with risk factors such as recent hospitalisation, indwelling catheters, prior MRSA colonisation, or treatment failure with standard regimens [26]. Once MRSA is confirmed, therapy should be narrowed to targeted agents, minimising collateral damage to the microbiome and resistance selection.

The role of rapid diagnostics, as highlighted in the 'Diagnostics of MRSA in UTIs' section, integrates directly with stewardship. PCR confirmation of MRSA enables early de-escalation in negative cases and timely initiation of targeted therapy in positive ones [31]. Current therapeutic options, their advantages, and limitations are outlined in Table 3.

**Table 3:** Treatment Options for MRSA Urinary Infections MRSA: methicillin-resistant *Staphylococcus aureus*; UTIs: urinary tract infections; MDR: multidrug-resistant; IV: intravenous; CPK: creatine phosphokinase

Therapy	Route	Advantages	Limitations	References
Vancomycin	IV	Reliable efficacy; widely available	Nephrotoxic; requires monitoring	[36]
Linezolid	IV/Oral	Excellent bioavailability; allows oral step-down therapy	Hematologic and neurologic toxicity (peripheral/optic neuropathy)	[36]
Daptomycin	IV	Potent bactericidal activity against MRSA	Not for pneumonia; monitor CPK levels	[36]
Faropenem (emerging)	Oral	Promising pediatric UTI therapy	Limited data available for MRSA infections	[38]
Biapenem (novel)	IV	Experimental carbapenem with potential anti-MRSA activity	Not standard of care; limited clinical validation	[23]
Amniotic extract (experimental)	Topical/Adjunct	Demonstrates alternative antibacterial effect against MDR uropathogens	Early-stage research; not clinically implemented	[39]
Supportive + surgical	-	Drainage or removal of infected devices; essential for source control	Requires invasive procedures	[33]

Surgical and supportive management

In complicated MRSA UTIs, particularly those associated with abscesses or obstructive uropathy, antimicrobial therapy alone may be insufficient. Surgical intervention, such as drainage of prostatic or renal abscesses, is essential for definitive management [33]. Similarly, catheter removal or replacement is a cornerstone of CAUTI management, as biofilms protect bacteria from antibiotic penetration [35]. Supportive care, including hydration and monitoring of renal function, complements antimicrobial therapy, especially in patients receiving nephrotoxic agents like vancomycin [36].

Future prospects

The treatment of MRSA UTIs is poised for evolution as new antimicrobials and adjunctive therapies are investigated. Agents such as sulopenem, advanced oxazolidinones, and non-antibiotic strategies (e.g., bacteriophage therapy, immune modulation) hold promise. The One Health approach, recognising the role of environmental and zoonotic reservoirs, may also inform preventive strategies to reduce the burden of resistant infections. Ultimately, the integration of novel therapies, stewardship, and preventive measures will determine the trajectory of MRSA UTI management in the coming decades.

Integrating local and global evidence

The prevalence of MRSA UTIs varies considerably across global and regional studies, reflecting differences in healthcare infrastructure, patient demographics, and antimicrobial practices. Comparative analyses show that MRSA prevalence among urinary isolates ranges from approximately 2-3% in multicentric Indian studies to 10-12% in Ethiopian hospitals and 7-9% in Saudi Arabia [6,14,24]. Despite these variations, the clinical implications remain consistent-MRSA UTIs, though relatively uncommon, pose significant risks of therapeutic failure and bacteremia if unrecognised or undertreated [12,34].

Globally, MRSA urinary infections often mirror resistance patterns seen in bloodstream and skin isolates. Reports of cefoxitin resistance as a surrogate marker for mecA expression [8], along with sustained susceptibility to vancomycin and linezolid in South Asian and pediatric populations [24,25], highlight the persistence of core resistance mechanisms. These findings underscore the need for continued surveillance and harmonised diagnostic strategies. Adopting a One Health perspective, encompassing human, animal, and environmental reservoirs, can further strengthen understanding of MRSA transmission dynamics and inform evidence-based infection control policies [11,13,21,22,32].

Clinical and public health implications

From a clinical perspective, even a modest prevalence of MRSA in urinary isolates necessitates vigilance. Empiric therapy for complicated UTIs in high-risk patients may need to account for MRSA, particularly in individuals with catheters, recurrent infections, or prior MRSA colonisation. At the same time, stewardship requires avoiding indiscriminate MRSA coverage in low-risk patients. Rapid diagnostics such as PCR can help balance these priorities by enabling targeted therapy [31].

Public health implications extend to infection control policies. Surveillance data from South Asia indicate that MRSA UTIs remain relatively uncommon, but lapses in catheter care or antimicrobial stewardship could allow for expansion. Evidence from studies demonstrating the discontinuation of contact precautions without increased MRSA rates [28] suggests that horizontal interventions, such as hand hygiene, decolonisation strategies, and device management bundles, may be more impactful than isolation alone.

Future directions

Future research should address several critical gaps in understanding and managing MRSA UTIs. Expanded surveillance through larger, multicenter studies across India and South Asia is necessary to more accurately define MRSA UTI prevalence and evolving resistance trends, while integration with global surveillance networks would enable benchmarking against international standards. Molecular epidemiology using genomic sequencing of urinary MRSA isolates could elucidate clonal lineages and trace relationships among community, hospital, and zoonotic strains, enhancing understanding of transmission dynamics.

In terms of novel therapeutics, reliance on a limited set of agents such as vancomycin and linezolid underscores the urgent need for alternatives. Continued research into newer agents such as faropenem [38], biapenem [23], or biologically derived therapies [39] is essential to strengthen the treatment arsenal. A One Health-integrated approach should also be prioritised to investigate MRSA reservoirs in food, livestock, and environmental sources, which may play significant roles in transmission to humans. Finally, stewardship interventions, including clinical trials assessing diagnostic-guided de-escalation for UTIs, similar to PCR-based protocols used for MRSA pneumonia [31], could minimise unnecessary broad-spectrum antibiotic use while maintaining effective and safe patient management.

## Conclusions

MRSA represents a small but clinically significant cause of UTIs worldwide. Published studies across diverse regions confirm the presence of MRSA among urinary isolates, even in settings where Gram-negative organisms predominate. These findings align with global trends of rising multidrug resistance and reinforce the need for ongoing surveillance, antimicrobial stewardship, and targeted diagnostics. MRSA urinary infections illustrate the convergence of host risk factors, bacterial adaptation, and systemic resistance challenges. Their clinical spectrum ranges from asymptomatic bacteriuria to invasive disease, with significant morbidity in vulnerable populations. While current therapies remain largely effective, the narrowing pipeline of new agents underscores the importance of stewardship and continued therapeutic innovation. Integrating regional and global evidence provides a comprehensive framework for addressing MRSA UTIs and highlights that even uncommon pathogens can reflect broader patterns in the global AMR crisis. A combined strategy of surveillance, stewardship, preventive interventions, and drug development is essential to mitigate the threat of MRSA in urinary infections.
